# Editorial: Reviews in molecular evolution of infectious agents and diseases

**DOI:** 10.3389/fmicb.2026.1812186

**Published:** 2026-03-10

**Authors:** Axel Cloeckaert, Antonio Battisti, Jens Andre Hammerl

**Affiliations:** 1 I NRAE, Université de Tours, UMR, ISP, Nouzilly, France; 2 Institute of Experimental Zooprophylactic of the Lazio and Tuscany Regions (IZSLT), Rome, Italy; 3 Department Biological Safety, German Federal Institute for Risk Assessment (BfR), Berlin, Germany

**Keywords:** antimicrobial resistance, bacteria, epidemiology, fungi, genetics/genomics, immune response, pathogenesis, pathology

The current Research Topic aims to publish high-quality scholarly review articles on key topics in molecular epidemiology and the evolution of infectious pathogens. It aims to highlight recent findings in the field while emphasizing important directions and new possibilities for future inquiries. Ten articles were published, consisting of six Reviews, two Systematic Reviews, one Mini Review, and one Author's Correction. The six Review articles address the molecular evolution of bacterial, viral, fungal, and parasitic pathogens. Li et al. provided a comprehensive overview of the genomic evolution of enteric pathogens and their mechanisms of pathogenicity and resistance to therapeutic agents. With regard to bacterial intestinal pathogens, the authors focused in particular on human pathogenic *Escherichia coli* pathotypes, *Klebsiella* spp., *Shigella* spp., and *Campylobacter* spp., their pathogenicity, and antimicrobial resistance mechanisms, including horizontal transfer of genetic determinants. The situation with gut fungi, mainly *Candida* spp., appears different, with adaptation occurring predominantly through genome remodeling and lineage-specific gene family diversification, rather than horizontal gene transfer (HGT) mechanisms central to bacterial evolution. As a consequence, antifungal resistance mechanisms arise essentially through point mutations and by gene amplification to enhance efflux pump and target enzyme expression. The pathogenic evolution of intestinal parasites, particularly protozoans (e.g., *Cryptosporidium* spp.), relies mainly on reductive genomic evolution, resulting in antigenic plasticity and modulation of host regulatory networks to establish chronic infections within the gastrointestinal tract. Genomic evolution of intestinal viruses (e.g., rotaviruses, noroviruses) is mainly driven by mutation, recombination, and immune selection to enhance their virulence, transmissibility, and zoonotic potential. Of note, in their review, the authors also reported on prion evolution, mediated solely through protein conformational transitions. This process is elaborately regulated by host genetic factors (notably PRNP polymorphisms) and the intestinal microenvironment. The authors also highlighted advances in the field of diagnosis (e.g., the development of precision diagnostics or therapy, including novel therapeutic strategies), taking into account the genomic evolution of enteric pathogens.

Two Review articles focused on specific bacterial pathogens. Li and Farzana reviewed mobile genetic elements (MGE) shaping *Klebsiella pneumoniae* pathogenicity. Among MGEs, plasmids, integrative conjugative elements (ICE), insertion sequences (IS), transposons, and integrons have contributed to the evolution of *K. pneumoniae* from an opportunistic pathogen toward hypervirulent strains causing severe infections in healthy individuals, with, in addition, carbapenem-resistant variants achieving high mortality rates. Mikhailovich et al. provided a current view on *Stenotrophomonas maltophilia* virulence. As with *K. pneumoniae*, this bacterial species has evolved from an opportunistic pathogen to a critical pathogen in the COVID-19 pandemic background, due to the fact that it is one of the most common causative agents of respiratory co-infections and bacteremia in critically ill COVID-19 patients. *S. maltophilia* adapts rapidly to unfavorable conditions, such as during antimicrobial treatment of hospitalized patients. Being initially intrinsically multidrug-resistant, under the selective pressure of hospital conditions, rapid acquisition of adaptive mutations can increase its resistance to many antimicrobials by upregulating its multiple efflux pump arsenal, and biofilm formation may also help it survive and persist in hospital environmental conditions. Moreover, these conditions appear interconnected to regulate the virulence mechanisms of *S. maltophilia*.

Two Review articles covered viral infections. Yu et al. provided a comprehensive overview of advances in research on severe fever with thrombocytopenia syndrome virus (SFTSV). This virus causes an acute infectious disease with a high mortality rate in severe human infections and has spread globally, in particular in Asian countries such as China, South Korea, and Japan. As with other pandemic viruses, SFTSV evolves rapidly through genetic mutations, reassortment, and homologous recombination. This evolution may affect pathogenesis and the rapid detection/diagnosis of the virus, along with the prevention of its spread. Wei et al., in a Mini Review, provided an update on feline calicivirus (FCV), comprising viral evolution, pathogenesis, epidemiology, prevention, and control. FCV is a prevalent and impactful viral pathogen affecting domestic cats. This virus displays high mutability and genetic plasticity, enabling its persistence within cat populations. Clinical manifestations range from asymptomatic infection and mild oral or upper respiratory tract infection to potentially virulent systemic and fatal infection. Persistence in the feline population is associated with concomitant viral genetic evolution resulting in altered immune responses and sequential reinfection. It potentially affects also its detection/diagnosis, prevention, and control.

With regard to infections caused by fungi, La Santrer et al. reviewed the protein kinase (PK) family in fungi about their adaptability, virulence, and conservation between species. This large family of enzymes plays a central role in cellular signaling through protein phosphorylation and controls cellular processes in response to stress conditions. The authors reviewed the role of kinases in fungal stress adaptation, discussing how the high conservation of their catalytic kinase domains makes them valuable as phylogenetic markers and therapeutic targets for several important fungal pathogens, such as *Candida albicans, Cryptococcus neoformans*, and *Aspergillus fumigatus*.

One Review article by Wu et al. focused on more technological aspects, namely Argonaute protein-based nucleic acid detection technology for rapid diagnosis of pathogens. Argonaute (Ago) proteins are recently discovered nucleases with nucleic acid shearing activity that exhibit specific recognition properties beyond CRISPR–Cas nucleases. The authors reviewed the current knowledge on the structure and function of Ago proteins and discussed the latest advances in their use for nucleic acid and pathogen detection.

Two articles of the Research Topic were Systematic Review articles, consisting of a literature search and analysis using several databases. Wang et al., by analyzing a selection of 27 studies, attempted to determine the molecular clock evolution rate of clinical *Mycobacterium tuberculosis* isolates. According to their analysis, the mutation rate of clinical *M. tuberculosis* strains is below 1 SNP per genome per year, indicating evolutionary stability in clinical settings. This finding appears important for tuberculosis outbreak reconstructions and public health strategies. Briki et al. performed a systematic search in literature databases over the period January 2010 to July 2025 to assess the molecular epidemiology of methicillin-resistant *Staphylococcus aureus* (MRSA) in Gulf Cooperation Council (GCC) countries. According to their analysis, MRSA in the GCC shows dynamic and evolving patterns. The authors propose that the One Health approach, combined with strengthened antimicrobial stewardship, mandatory hospital screenings, and wastewater monitoring, could improve MRSA detection, tracking, and control across the region.

In summary, the contributions to the present Research Topic provide an overview of current knowledge on the molecular evolution of several infectious pathogens of importance in human or veterinary medicine. We hope it will promote discussion in the infectious disease community that will translate to best practice applications in clinical, public health, and policy settings.

